# DNA molecule stretching through thermo-electrophoresis and thermal convection in a heated converging-diverging microchannel

**DOI:** 10.1186/1556-276X-8-87

**Published:** 2013-02-18

**Authors:** Shou-Shing Hsieh, Jyun-Hong Chen, Cheng-Fung Tsai

**Affiliations:** 1 Department of Mechanical and Electromechanical Engineering, National Sun Yat-Sen University, Kaohsiung, 80424, TaiwanRepublic of China

**Keywords:** single DNA molecule stretching, CLSM, thermo-electrophoresis, converging–diverging microchannel

## Abstract

A novel DNA molecule stretching technique is developed and tested herein. Through a heated converging-diverging microchannel, thermal convection and thermophoresis induced by regional heating are shown to significantly elongate single DNA molecules; they are visualized via a confocal laser scanning microscopy. In addition, electrophoretic stretching is also implemented to examine the hybrid effect on the conformation and dynamics of single DNA molecules. The physical properties of the DNA molecules are secured via experimental measurements.

## Background

The past two decades has witnessed a tremendous growth in knowledge regarding the mechanical properties of DNA and its polymeric behavior. In addition, developments in molecular biology and micro- or nanotechnology have increased the interest of scientists and engineers in the mechanical manipulation of single DNA molecules. In fact, engineering DNA stretching would be a key step in the development of the next generation of biological microfluidic devices [1].

The ability to directly manipulate and visualize single DNA molecules has led to a number of advances in our current understanding of the physical and biological properties of DNA. Two general approaches to DNA stretching are in common use: DNA is stretched in a solution as it flows through a microchannel or it is stretched on a solid surface. Both approaches have their own advantages/disadvantages which depend on the particular application. For the former, with fluorescently labeled DNA molecules, it is possible to visualize the change in the conformation of a single DNA molecule under an optical microscope [2,3].

Recently, Ichikawa et al. [4] have presented a novel DNA extension technique via laser heating. They proved that the new stretching technique was promising and could work in selected applications. Thermophoresis has also been found to play an important role in DNA molecule stretching.

The thermal convection induced in this study was similar to the convection that is inferred for the well-known Earth's mantle convection/or Bernard cell convection. Such convection produced the horizontal flow which caused the movement of the solution. Following [4], the governing equations of thermal convection in the study are the conservation equations of mass, momentum, and energy with the major dimensionless parameter of the Rayleigh number, indicating the vigor of convection and nondimensionalized heat flux.

**Figure 1:**
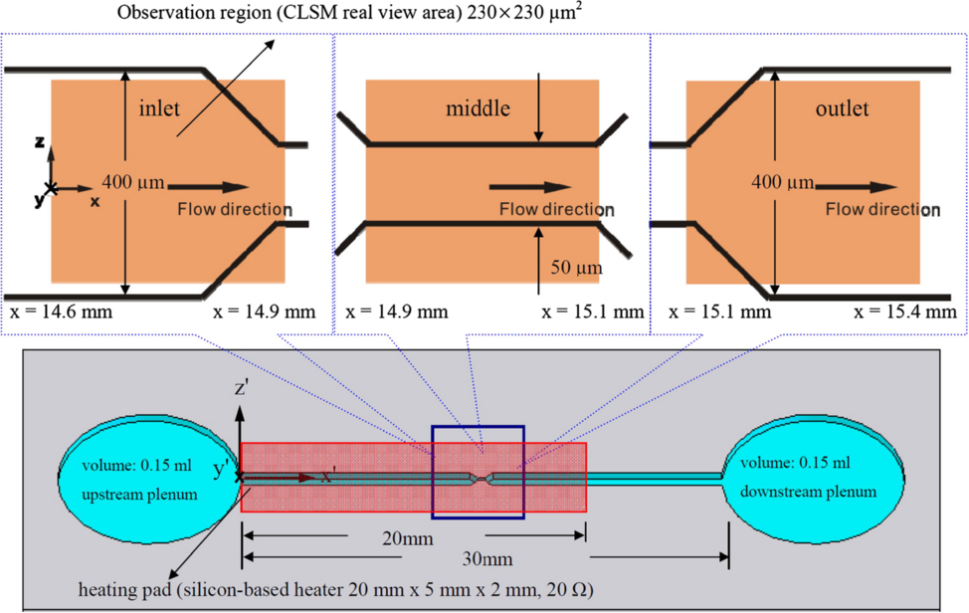
Microchannel geometry and observed sections.

In this study, a more powerful modified method was used to stretch a single DNA molecule in an electro-osmotic flow (EOF) through a heated converging-diverging microchannel, using both confocal laser scanning microscopy (CLSM) and micro-particle image velocimetry (μPIV) measuring/visualization techniques. Figure 1 shows schematically the gradual contraction (8:1)/gradual expansion (1:8) flow cell system used in this study. Our main focus was to examine the contribution of stretching due to thermal convection, thermophoresis, electrophoresis, or a combination thereof in order to gain further insights into the flow behavior of the DNA stretching mechanism and the physical/mechanical properties of single DNA molecules, as well as related phenomena.

## Methods

### PDMS flow cell fabrication

For this study, we used a 400 × 50 μm and 50 × 400 μm converging-diverging test section with a heating foil, which is a silicon-based heater with a size of 20 × 5 × 2 mm, with a total electrical resistance of 20 Ω, connected to a direct current (DC) power supply (N6731B DC power supply module) embedded underneath the backside of the floor of the channel. The size and dimensions of the heating foil were chosen and designed so that the temperature distribution on the *xz* plane (at *y* = 0) of the test section remained uniform upon heating. The microfabrication process followed that of [3], except for slight modifications in the channel size and converging-diverging ratio. The relevant geometric size and dimensions are listed in Table 1. After completing (8:1:8) the fabrication, the test channels were rinsed in acetone and ethanol and dried with an argon stream. The present study used untreated/treated polydimethylsiloxane (PDMS) channel to measure electrophoresis (DNA molecules) velocity and total velocity of EOF, respectively.

**Table 1:** Relevant parameters

**Parameters**			**Value**		
Channel total length, L_t_			30 mm		
Channel test section length, L_s_			0.66 mm		
Channel contraction length, L_m_			0.2 mm		
Channel main width, W_m_			0.4 mm		
Channel contraction width, W_c_			0.05 mm		
Channel depth, H			0.1 mm		
Channel hydraulic diameter, D_h_			66.67 ~ 160 μm		
Channel contraction ratio			8:1		
Channel expansion ratio			1:8		
Electric field (kV/m), E_x_			5, 7.5, 10		
DNA concentration, μg/ml			0.065		
Working fluid			1x TBE		
Viscosity (cP), μ			1 cP		
Reynolds number, Re			0.032 ~ 0.064		
λ-DNA contour length (μm) (labeled with YOYO-1)			21		
Radius of λ-DNA gyration (μm)			0.7		
Temperature ( C), T	25	35		45	55
Relaxation time (s), τ_r_ (Rouse model)	0.0456	0.0441		0.0427	0.0414
Relaxation time (s), τ_e_ (Experiment)			0.6		
Deborah number			1.2 ~ 2.3		
Velocity vector distribution					

For the tested channels, precise information on the channel dimensions was extremely important in order to make an accurate evaluation. The depth, width, and length were measured optically within an accuracy of ±0.2%. To understand the surface condition of the present device, the roughness of the channel was measured along its center with a surface profilometer.

Each of the reservoirs (up/downstream plenum) had a volume of 0.15 ml. The channel had a total length of 30 mm, with a length of 800 μm for the test section. The detailed values of the test cells are listed in Table 1.

### CLSM/μPIV and μLIF setup

**Figure 2:**
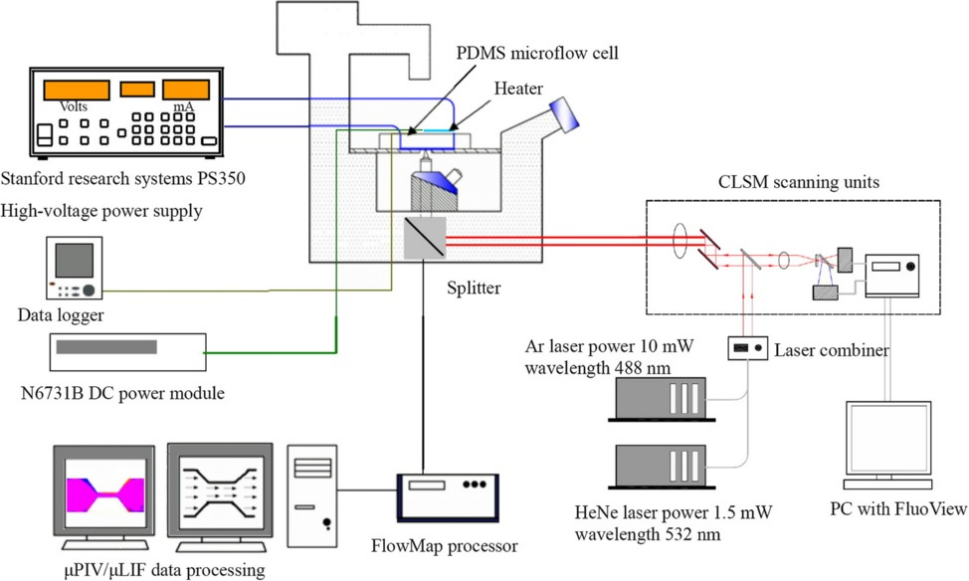
Schematic of the CLSM/ instrumentations.

**Figure 3:**
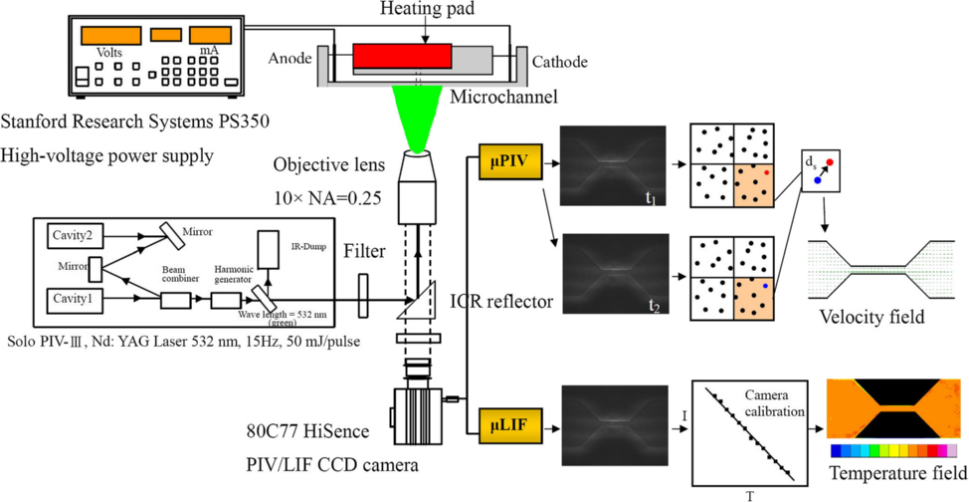
Schematic of the μPIV/laser-induced fluorescence (μLIF) system velocity and concentration measurements.

The CLSM measurement setup, as shown in Figure 2, is combined with a laser light source (Ar-ion laser 488 nm/ HeNe laser 532 nm) and scanning system in order to generate the entire field. The flow cell was mounted onto an epifluorescent microscope (IX71/FV300, Olympus, Tokyo, Japan) equipped with a ×40 magnification, NA 0.85 air immersion objective lens, following that described by [3]. The EOF was driven by a high-voltage power supply (PS 350, Stanford Research System, Sunnyvale, CA, USA) to drive the flow, with a slight modification for the flow cell and the flow circulation loop. For that reason, all the details have not been repeated here. The experimental scheme used to implement the μPIV measurement is shown in Figure 3. The use of the μPIV technique is very attractive in microfluidics because it helps to determine the detailed flow phenomena of microsystems by utilizing flow-tracing particles to map the flow in the microchannels. In this study, the stained DNA molecules could also be used as seeding.

The setup shown in Figure 3 was based on two pulsed Nd:YAG lasers (New Wave SoloII, New Wave Research, Fremont, CA, USA; 30 mJ, double cavity) firing on the second harmonic SoloII (green, 532 nm). The laser provided a laser beam with a measured area. The light was positioned so as to illuminate the entire inlet, outlet, and midsection of the channel. The laser pulse duration was 4 to 80 ms, based on the velocity magnitude. The test system was mounted on a movable *xz* stage on an inverted epifluorescence microscope (DMILM, Leica, Solms, Germany) with ×10 magnification, 0.25-numerical aperture panchromatic objective, and a field view of 800 × 600 μm. The measurement plane (i.e., the object plane) was precisely positioned relative to the test section by vertically moving the objective lens in the *y* direction and by horizontally moving the table in the *x* and *z* directions. The concentration of stained DNA molecules based on the interrogation volume was 8 × 10^7^ particles/ml.

The images were recorded using a Dantec 80C77 HiSense PIV (Dantec Dynamics, Ulm, Germany) 1,344 × 1,024 × 12 bit interline transfer camera. Five images were taken for each flow field, with a spatial resolution of 32 × 32 pixels. The interrogation cell overlay was 50%. Background-noise influence was removed by subtracting the background intensity from the captured images. In addition, an ensemble averaging 20 images consecutively captured for 4 s was used to obtain the velocity measurements. The calculated measurement depth of the present μPIV was 50 μm. Each measurement was repeated at least three times under specified conditions. The measurements were conducted in the middle region at both the inlet and exit regions of the microchannel. The flow was found to have reached full hydrodynamic development at the middle region of the microchannel.

Visualization of the local buffer solution temperature was achieved with the same apparatus used for flow visualization and measurements (see Figure 3). However, instead of using stained DNA molecules, the channel was filled with a solution of rhodamine B, a fluorescent dye which shows a temperature-sensitive quantum yield in the range of 0°C to 100°C [5,6]. Experiments were conducted with a fluorescence microscope equipped with a long-working distance ×10 objective lens. The images were recorded with the same equipment used for the μPIV measurements. From the captured images, the detailed temperature distribution could be extracted. Following [5], the intensity values of the captured images were converted to temperature using intensity-versus-temperature calibration; calibration of the intensity of temperature was made for each solution.

### Flow system

In the electro-osmotically driven flows, a 30-mm-long converging (8:1)-diverging (1:8) microchannel with a cross section of 100 × 400 μm and two reservoirs (up/downstream plenum) was used to supply a buffer of stained DNA molecules for the channel. Before use, the microchannel and entire flow loop were rinsed with DI water for at least 1 h to remove any contaminants. The transparent nature of the microchannel surfaces allowed visual examination of the channels to ensure that no bubbles were left. The buffer solution used was 1× Tris-borate with ethylenediaminetetraacetic acid (EDTA) (TBE) with pH 8.3. A schematic diagram showing the flow cell and the auxiliary system is given in Figure 3.

During each measurement, the microchannel was connected to small reservoirs. Current data were recorded from the power source by a personal computer-based data acquisition system. μPIV measurements were taken through a viewing window at midplane (*y* = 0) between the two cylindrical reservoirs with a diameter of 5 mm. The potential was applied via platinum electrodes immersed in the two 0.15-ml open reservoirs. The distance between the two reservoirs was 30 mm. When electric field was >10 kV/m, the EOF velocity of the solution will increase, and the mobility would be dependent on the electric strength [6,7]. In order to avoid joule heating, electric field strengths of 5, 7.5, and 10 kV/m were thus applied.

The μPIV measurement system included visualization and the capture of images, the calculation of two-dimensional velocity vectors, and post-processing for data analysis. The vector field of the flow velocity within the measurement plane of the light sheet was determined by measuring the displacement of the tracer particles and the time durations of two laser pulses. A PIV 2100 processor (Dantec Inc.) was optimized to process the μPIV images into a raw vector map in real time and to transfer the map to a database in the PC. The processor employed cross-correlation to calculate the velocity vectors. A total of 800 sets of data was taken at each location for a specified Reynolds number (Re; i.e., the ratio of inertial forces to viscous forces). The selection of 800 data sets was based on the examination of the data convergence. One set of data consisted of five PIV vector data for a 32 × 32 pixel interrogation area. These data were statistically averaged, and the mean vector fields were obtained and used for the examination of the flow structure. The measurements were performed in a clean room at the University Microsystem Laboratory at a controlled ambient temperature of 298 K.

### Methodology used (for electrophoretic mobility of DNA molecules and buffer solution EOF velocity) and temperature visualization

Following [7], the electrophoretic velocity of the stained DNA molecules in the untreated PDMS channel with negligible electro-osmotic mobility was measured using μPIV measurements. The total velocity of the seed particles (i.e., DNA molecules) can also be measured through the μPIV measurements for treated PDMS channels. With these velocities found, the bulk averaged EOF velocity of the fluid (*u*) could be obtained following equation (1) below:

(1) $$u=\;{\bar{u}}_{m}-\;{\bar{u}}_{\mathrm{ph}}$$

where ${\bar{u}}_{m}$ is the total velocity of the seed particles (i.e., DNA molecules) by μPIV in treated PDMS channels, and ${\bar{u}}_{\mathrm{ph}}$ is the electrophoretic velocity of the DNA molecules in the untreated PDMS channel.

With respect to measurement uncertainties, the most significant source of error was expected to be the measurements at the wall, and the biggest physical error in the μPIV data was the Brownian diffusion of the stained DNA molecules. Out-of-plane Brownian diffusion causes a reduction of the signal-to-noise ratio of the cross-correlation peak, and such an error was estimated. Errors due to in-plane Brownian diffusion were essentially eliminated by temporally averaging the results in the steady flow. In fact, experimental errors due to the limiting spatial resolution of the CCD camera, as well as errors in determining magnification, were therefore the major source of error in these results and found to be within ±15%.

Visualization of the local fluid temperature was achieved with the same apparatus used for flow visualization and measurements (see Figure 3). Instead of using fluorescent particles, however, the channel was filled with a solution of rhodamine B, a fluorescent dye which shows a temperature-sensitive quantum yield in the range of 0°C to 100°C [5]. Experiments were conducted with a fluorescence microscope equipped with a long-working distance ×10 objective lens. The images were recorded with the same equipment used for the μPIV measurements. From the captured images, the detailed temperature distribution could be extracted. The intensity values of the captured images were converted to temperature using the intensity-versus-temperature calibration [5]. A calibration of the intensity of temperature was made for each solution.

### Sample preparation

On the basis of standard lithography techniques, we constructed a 30-mm-long, 400-μm-wide, and 100-μm-high PDMS microchannel with a sudden contraction/expansion (a ratio of 8:1:8) test section 20 mm in length. Reservoirs (4 × 4 mm) were cut at each end of the curved PDMS microchannel with a scalpel, and the channels were soaked for 12 h at 45°C in 1× TBE (1× TBE contains, in 1 l, 108 g of Tris base, 55 g of boric acid, and 40 ml of 0.5 M EDTA, pH 8.3) to eliminate permeation-driven flow [3].

λ-phage double-strand DNA (dsDNA) from New England Biolabs (Ipswich, MA, USA) was used as the tracer in the present study. The DNA was stained, with respect to the backbone, with a fluorescent dye (YOYO-1, 4.7:1 bp/dye molecule), for a total length of 48.5 kbp DNA molecules, and diluted in 1× TBE. The dyed λ-DNA had a contour length (*L*_c_) of 21 μm [3], and the longest relaxation time (*τ*_e_) of 0.6 s (from uncoiled maximum length to coiled state) was measured and found in the present study.

## Results and discussion

### DNA molecule velocity profile with/without temperature effect

**Figure 4:**
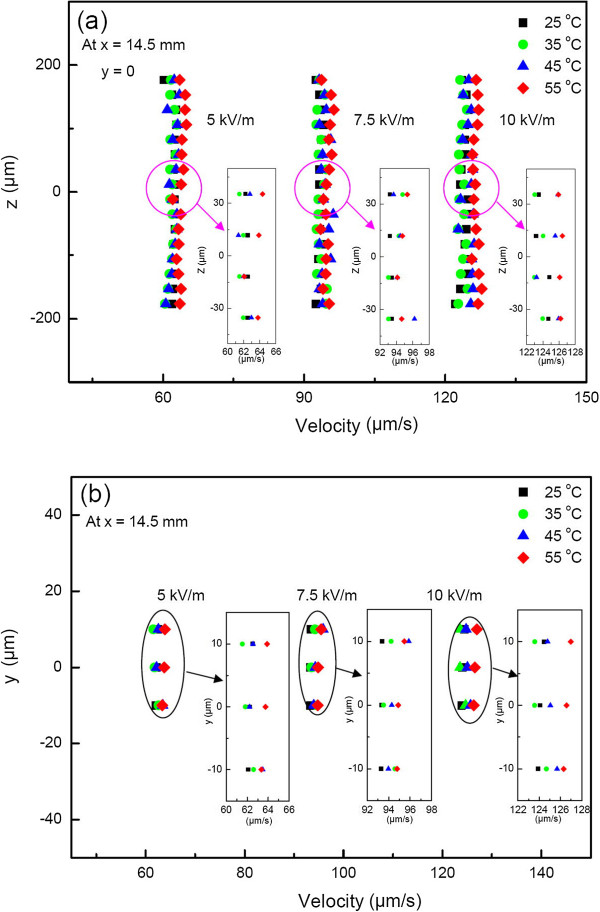
**DNA molecule velocity at different heating temperatures and electric strength at the channel inlet.** (**a**) Spanwise (*x* = 14.5 mm) and (**b**) transverse (*x* = 14.5 mm).

Spanwise velocity profiles of DNA molecules at *y* = 0 in 1× TBE buffer at the inlet regions (*x* = 14.5 mm) of the *D*_h_ = 160 μm microchannel at *E*_x_ = 5, 7.5, and 10 kV/m without joule heating are given in Figure 4a. The plug-like motion, a characteristic of an electrokinetic-driven flow, was apparent, and the velocity profiles remained fairly flat right to the wall for *E*_x_ ≤ 10 kV/m. On the other hand, the streamwise velocity profiles (not shown) of DNA molecules along the downstream at the inlet regime of the channel exhibited a nearly mountain-like distribution, similar to those reported in [3] for EOF with different magnitudes. The differences of about one order of magnitude were due to the former being electrokinetic driven, while the latter was pressure driven. In addition, the former was for DNA molecules along the downstream velocity, while the latter was for the EOF velocity of the buffer solutions. Nonetheless, they had the same developing trend, and they all increased as the *E*_x_ increased. Figure 4b shows the corresponding transverse velocity distribution. Likewise, the similar plug/uniform velocity profile again appeared. The insets in Figure 4a,b were made for clarity. Although the plug/uniform velocity distribution in the *y* and *z* directions was what one would expect without the joule heating effect, very small velocity differences in both the *y* and *z* directions were still noted upon close examination as the buffer solution was heated to different temperatures of 25°C, 35°C, 45°C, and 55°C. In addition, the velocity discrepancy increased as the heating temperature increased in both the *y* and *z* directions.

Taking a further look at Figure 4a,b, with a working temperature of 25°C, the DNA molecule velocity had an electrophoretic velocity apparently of the same order of magnitude in both the *y* and *z* directions due to the uniformity across the stream. The velocity was constant across the channel width (along the *z* direction) and height (along the *y* direction) with an increase from 60 to 125 μm/s as *E*_x_ = 5 kV/m increased to 10 kV/m, as shown in Figure 4a,b. Tables 2 and 3 list the local velocity distributions measured at different electric strengths and heating temperatures, respectively.

**Table 2:** Local velocity map (μm/s) with different heating temperatures

***y *(μm)**	**25**º**C**			**35**º**C**			**45**º**C**			**55**º**C**		
10	62.51	93.40	124.45	61.55	94.23	123.59	62.55	95.88	124.79	63.89	95.46	126.97
0	62.23	93.33	124.09	61.83	93.53	123.57	62.22	94.29	125.06	63.74	94.89	126.57
−10	62.10	93.30	123.88	62.61	94.59	124.68	63.48	93.98	125.68	63.35	94.79	126.30
Error (%)	0.66	0.11	0.46	1.72	1.13	0.9	2.03	2.02	0.71	0.85	0.71	0.53

Velocity map at different heights of the channel in *x* = 14.5 mm and *y* = 10 to −10 μm.

**Table 3:** Local velocity map (μm/s) with different heating temperatures and electric fields

***T *( C)**	**5 kV/m**			**7.5 kV/m**			**10 kV/m**		
25	62.23	62.31	62.52	93.33	93.44	93.53	124.09	124.05	124.20
35	61.83	62.45	62.56	93.53	93.55	93.60	123.57	123.78	123.94
45	62.22	62.33	62.54	94.29	93.88	93.90	125.06	124.99	125.15
55	63.74	63.54	63.60	94.89	94.67	94.75	126.57	126.41	126.43
Error (%)	3.10	1.97	1.73	1.67	1.32	1.30	2.42	2.12	2.01

Velocity map at different locations of the channel in *x* = 14.5, 14.6, and 14.7 mm and *y* = 0.

**Figure 5:**
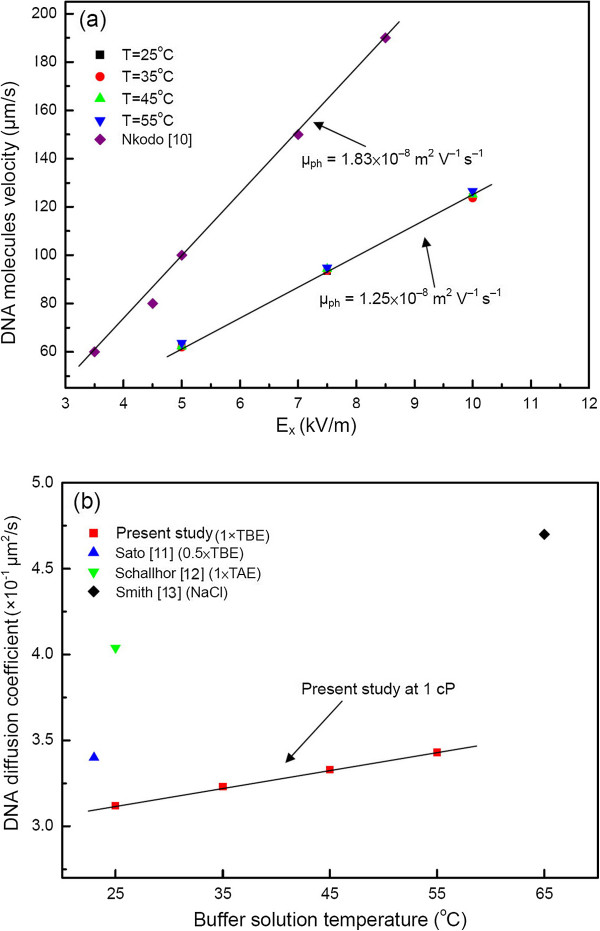
**Relative velocity of the buffer solution convection.** Velocity gradient at different electric fields and at a definite channel inlet *x* = 14.5 mm (**a**, **b**, **c**) and different channel velocity profile (**d**, **e**, **f**) at *y* = 0 at different channel positions (a, b, c) with different heating temperatures and electric strengths.

Figure 5 shows the relative velocity (|ΔV|; absolute value was taken), using a treated PDMS device to get the velocity of EOF of the buffer solution convection observed at four different temperatures, 25°C, 35°C, 45°C, and 55°C, for four corresponding heating powers at three electric strengths. Convection rates were estimated by the increases in buffer solution temperature. Two different trends were observed: one (left half) at the same inlet position with different elevation and the other (right half) at the same elevation with a different downstream position. The former showed an irregular velocity |ΔV| distribution as the heating temperature increased at different electric strengths, while the latter exhibited a definite quadratic |ΔV| increase as the heating temperature increased. The significant influence of the buffer solution temperature increase on DNA molecule stretching was clearly noted. This was partly because the rise in temperature of the buffer governed the evaporation rate which would result in suspension deposited on the channel bottom surface, and consequently, it created the convective flux that dragged the DNA molecules and resulted in molecule stretching. Therefore, we concluded that thermal convection or thermophoresis caused DNA molecule elongation. In addition, with such temperature differences in the buffer solutions, certain effects due to thermal diffusion might have also occurred, which will be discussed later.

Again, Figure 5 shows the velocity of the buffer solution convection observed for four different heating temperatures at the up, middle, and downstream locations, respectively (right half). The convection rates were approximately linear with the heating power and coincided with those found in Mao et al. [8], but they were strongly affected by the location where the velocity was measured. It was found that the convection effect became more dominant as the flow proceeded downstream, which was in good agreement with those of the temperature distributions, namely, the temperature gradient became steeper downstream than upstream.

### DNA electrophoretic mobility and diffusion coefficient

**Figure 6:**
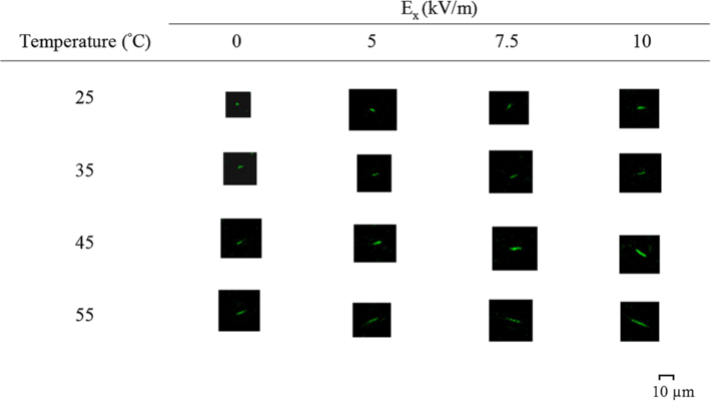
**DNA molecule mobility and diffusion coefficient distribution.** (**a**) DNA electrophoresis velocity versus electric field and (**b**) relationship of diffusion coefficient and buffer solution temperatures [11-13].

Electrophoresis is the net migration of a molecule induced by Coulomb forces on a charged molecule or particle. Despite the complexity of the physics that governs DNA electrophoresis, based on the above-stated velocity results, the electrophoretic mobility of long DNA in the buffers was found to be in the range of *μ*_ep_ = 1.25 × 10^−8^ m^2^/Vs, which was in good agreement at a same order (approximately 10^–8^) with [9]. Note that the thermophoresis effect in the calculation was neglected here for simplicity. Figure 6a shows the electrophoretic mobility of the DNA molecules. Generally, distribution is a linear function of a velocity-versus-electric field strength graph. In this figure, the slope of the lines represents the electrophoretic mobility, *μ*, with a close-up view of *μ* at different temperatures. The temperature effect is not clearly noted. Again, this indicates that thermophoresis can be neglected. Furthermore, the results from [10] were with ssDNA, which has a smaller molecular weight than the DNA molecules used in the present study. Thus, there was a much higher mobility of *μ*_ph_ , as depicted in Figure 6a.

Diffusion in the present study could be classified as translational diffusion or rotational diffusion. Only translational diffusion, i.e., diffusion of the center of the mass of DNA molecules, was considered. The translational diffusion was proportional to the thermal energy and, thus, proportional to *k*_B_*T*, as well as the effective viscous mobility, *μ*. Following [8], we approximated the DNA diffusion coefficient as *D* = *k*_B_*T* /6π*ηr*_g_, where *η* is the buffer viscosity, *r*_g_ is the gyration radius of the DNA molecule, and *k*_B_ is the Boltzmann constant, which was 3.12 to 3.43 × 10^−1^ μm^2^/s in the temperature range of 25°C to 55°C, as shown in Figure 6b. Further comparisons were made with those of previous studies for *μ*_ep_ and diffusion coefficient *D*, and the results are shown in Figure 6a,b, respectively. Given the different buffer solutions at different temperatures and the shorter gyration radius of the present study, as expected, the diffusion coefficient *D* was lower, as illustrated in Figure 6b.

### Heating effect on DNA molecule stretching

Using detailed μLIF observations, thermophoresis, often called the Ludwig-Soret effect (thermal diffusion), was considered [14]. The investigation of the Soret effect in the buffer solution was based on the determination of the following transport coefficient: *D*_md_, mutual diffusion coefficient; *D*_T_, thermal diffusion coefficient; and *S*_T_, Soret coefficient. Detailed calculation of the values of the above-stated parameters improved our basic understanding of the exact stretching mechanisms involved in this study. However, due to the limitation of the measurements, several physical quantities above were not available at this stage. Further study could include this aspect. Nevertheless, thermal convection, as well as diffusion, was still noted.

**Figure 7:**
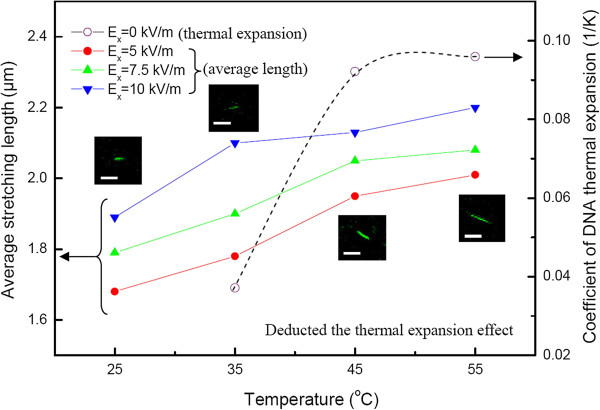
**Sample images of DNA molecule stretching.** With various temperatures and electric field strengths at the inlet region (*x* = 14.6 to 14.9 mm) via CLSM.

**Figure 8:**
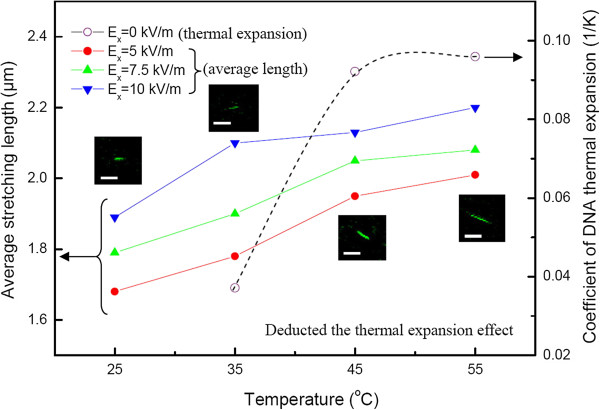
**Average stretching length.** After deducting the thermal expansion effect and coefficient of DNA thermal expansion versus temperature at the inlet region (14.6 to 14.9 mm) with the scale bar of 10 μm.;

Figure 7 shows these results at different streamwise electrical strengths without the joule effect (≤10 kV/m) at different temperatures. Note that thermal expansion occurred at *E*_x_ = 0. There were two groups with a similar developing tendency but different rates of increase: one at a heated temperature between 25°C and 35°C and the other between 35°C and 55°C, with two different slopes. Obviously, the latter had a greater heating effect than the former as far as the stretching length was concerned. For all the electric strengths studied, the trend of the development of stretching versus temperature appeared to be similar. After deducting the thermal expansion length, the DNA molecule average stretching lengths were found, and they were plotted against applied electric fields, as shown in Figure 8. The most significant stretching happened at *E*_x_ = 10 kV/m as the heating temperature increased from 35°C to 55°C. The effect of electric strength that deducted the thermal effects was also as expected, although the rate of increase was minimal. As stated previously, Figure 8 also shows the thermal expansion distribution (*E*_x_ = 0 kV/m) with different buffer temperatures. In addition, it was apparent that after the temperature rose to 45°C, the DNA molecule thermal expansion coefficients appeared to be independent of temperature and reached a constant at about 0.097 K^−1^.

### Heating effect on the histogram of DNA stretch ratio

**Figure 9:**
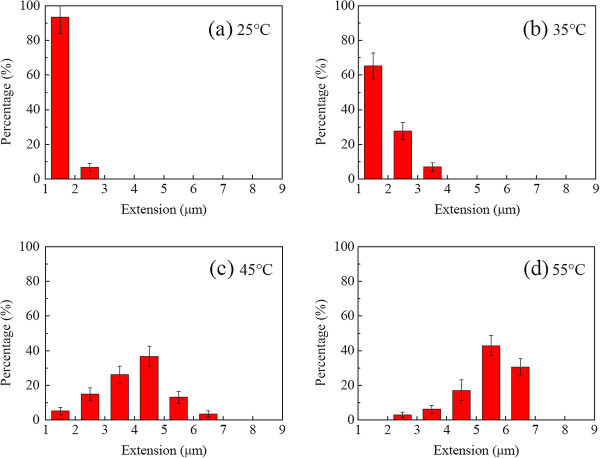
**Histogram of DNA length without electric field strength at different temperatures.** (**a**) 25°C, (**b**) 35°C, (**c**) 45°C, and (**d**) 55°C.

Figure 9 shows the DNA histogram of the stretch ratio without the electric field applied at the inlet region. The heating effect was clearly noted as the maximum extension length went from about 2.5 μm at 25°C to 6.5 μm at 55°C. In addition, 85% of the DNA molecules (≃85%) were at 1.5 μm at 25°C versus 40% at 5.5 μm, even with no external electric field employed. The stretching was partly due to thermal expansion of the DNA molecules (≤10%) and partly because of thermophoresis (≥90%). Each contribution (10% versus 90%) can be calculated based on a measured thermal expansion coefficient in Figure 8 and obtained.

**Figure 10:**
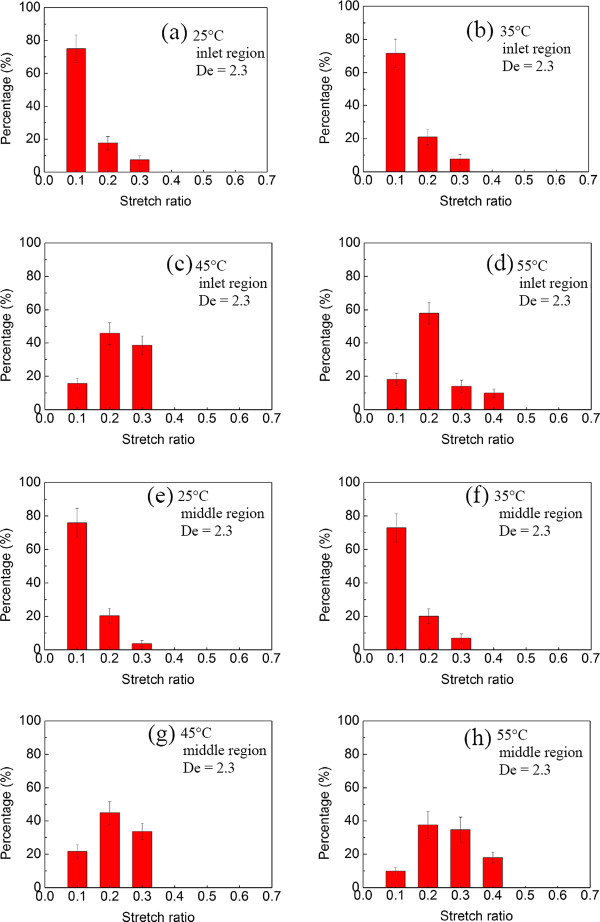
**Histogram of the stretch ratio of DNA molecule after deducting the thermal expansion effect.** At *E*_x_ = 10 kV/m at different temperatures. Inlet region: (**a**) 25°C, (**b**) 35°C, (**c**) 45°C, and (**d**) 55°C. Middle region: (**e**) 25°C, (**f**) 35°C, (**g**) 45°C, and (**h**) 55°C.

Moreover, when electric strength was applied, the stretch ratio was enhanced. Figure 10 shows respectively the corresponding results at different regions (inlet/middle) with different temperatures at *E*_x_ = 10 kV/m and Deborah number (De) = 2.3. The effect of the position either at the inlet/or middle region can be seen. At the downstream middle region, the DNA molecules seemed to be further stretched, and most significantly, more DNA molecules were found at a larger stretch ratio, for instance, 10% (inlet) versus 20% (middle) at 55°C and De = 2.3 for a stretch ratio of 0.4.

### Stretching force distribution

**Figure 11:**
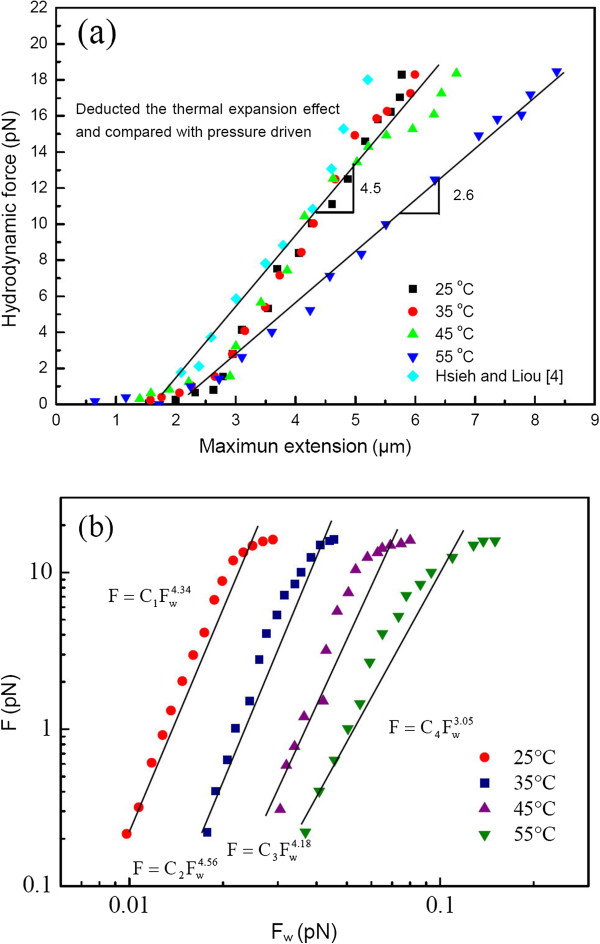
**Stretching portions of the force-extension curves as a function of temperature.** (**a**) Maximum DNA molecule hydrodynamic force versus extension after deducting the thermal expansion effect. (**b**) Hydrodynamic force of the present study versus the force law from the WLC model.

Extracting the data from Figure 10, the maximum extension distribution was deduced to be a function of the stretching force. The stretching portions of the force-extension curves as a function of temperature are shown in Figure 11, in which the DNA molecule maximum extension length versus hydrodynamic force after deducting the thermal effect can be drawn and compared with those from the well-known force law of the wormlike-chain (WLC) model. The stretching force clearly decreased as the temperature increased due to thermal convection and/or thermophoresis, as evidenced by the thermal convection velocity distributions, as shown in Figure 4b and especially in Figure 5a,b,c,d,e,f. With the thermal expansion effect deducted, the different temperature results were shown in Figure 11a. As expected, the temperature effect had a significant influence on extension. Unlike those in Hsieh et al. [2] or Hsieh and Liou [3], the present stretching behavior at a temperature of 55°C changed following the evolution of double strand, transition, and single strand, based on CLSM *in situ* observation. Even so, similar linear dependence behavior was still found with different slopes. In fact, as the temperature increased from 25°C to 45°C, the force-stretching length relationship was similar to that in Hsieh and Liou [3], as the comparison in Figure 11a shows. In addition, there was good agreement with almost equal slopes as the temperature increased from 25°C to 45°C. This finding also verified the correctness of the present measurement.

As Figure 11b shows, the present experimental data could be approximately fitted by applying the well-known WLC model. The hydrodynamic force measured and calculated through the Stokes formula was found to be a power law function of the forces via the WLC model with different exponents of 3.05 to 4.56 and different coefficients (C_1_ to C_4_) with different temperatures. Obviously, the stretching forces were greater than those predicted by the WLC. Furthermore, the temperature effect was again noted; otherwise the exponent found would have been nominally the same.

## Conclusions

DNA molecule dynamics in gradual/sudden converging-diverging heated microchannels were extensively examined via CLSM visualization and μPIV velocity measurements of dsDNA molecules in solutions at different temperatures, i.e., 25°C, 35°C, 45°C, and 55°C. The important points from this study were as follows:

1. A decrease in the stretching force was observed as the solution temperature increased, which was in good agreement with that in Williams et al. [15].

2. Although thermophoretic stretching was not clearly noted, the effect still seemed to occur and to increase as the temperature increased.

3. In addition to electrophoretic stretching, thermal convection made an equal contribution in terms of DNA molecule stretching.

4. As a result of points 2 and 3, when the buffer solution temperature increased, the stretch ratio was three to four times that of the isothermal buffer solution.

5. DNA molecule thermal expansion played a significant role (≥50%) in DNA molecule stretching. Therefore, the present stretching mechanism included thermal expansion, thermal diffusion (thermophoresis), and thermal convection.

6. Electrophoretic mobility and the translational diffusion coefficient of the DNA molecules were obtained and compared with those of existing data.

## Abbreviations

CLSM: confocal laser scanning microscopy; EOF: electro-osmotic flow; μPIV: micro-particle image velocimetry; μLIF: laser-induced fluorescence.

## Competing interests

The authors declare that they have no competing interests.

## Authors’ contributions

SSH provided the idea and drafted the manuscript. CFT was responsible for carrying out the experimental work and the basic result analysis. JHC helped design the experiment and assisted with the result analysis. All authors read and approved the final manuscript.

## Authors' information

SSH is a professor at the Department of Mechanical and Electro Mechanical Engineering, National Sun Yat-Sen University, Kaohsiung, Taiwan, Republic of China. JHC is currently working towards a PhD degree at the Department of Mechanical and Electro Mechanical Engineering, National Sun Yat-Sen University, Kaohsiung, Taiwan, Republic of China. CFT is a student working towards a master's degree at the Department of Mechanical and Electro Mechanical Engineering, National Sun Yat-Sen University, Kaohsiung, Taiwan, Republic of China.
